# InterProScan 6: a modern large-scale protein function annotation pipeline

**DOI:** 10.1093/bioadv/vbag141

**Published:** 2026-05-21

**Authors:** Matthias Blum, Emma Hobbs, Laise Florentino, Alex Bateman

**Affiliations:** European Molecular Biology Laboratory, European Bioinformatics Institute (EMBL-EBI), Hinxton, CB10 1SD, United Kingdom; European Molecular Biology Laboratory, European Bioinformatics Institute (EMBL-EBI), Hinxton, CB10 1SD, United Kingdom; European Molecular Biology Laboratory, European Bioinformatics Institute (EMBL-EBI), Hinxton, CB10 1SD, United Kingdom; European Molecular Biology Laboratory, European Bioinformatics Institute (EMBL-EBI), Hinxton, CB10 1SD, United Kingdom

## Abstract

**Motivation:**

InterProScan is a widely used software package for large-scale protein function classification and an essential component of UniProt, Ensembl and MGnify Genomes large-scale annotation pipelines. For the past 12 years, InterProScan 5 has provided robust analysis but faced challenges in scalability, installation, data management, and integration with modern workflow systems. We present InterProScan 6, a complete reimplementation as a Nextflow pipeline that enhances scalability, portability, and reproducibility across diverse computational environments, including local, HPC, and cloud platforms. Key improvements include decoupling of application code from signature data, native container support, on-demand data management, integration of modern deep-learning-based predictors, and a redesigned Matches API for retrieving pre-computed annotations in JSON format.

**Results:**

Across nine reference proteomes ranging from bacteria to complex eukaryotes, InterProScan 6 consistently reduced wall-clock runtime relative to InterProScan 5, with speedups of approximately two-fold on large eukaryotic proteomes. When pre-computed annotations were available via the Matches API, runtimes were reduced to minutes. Comparison of annotations generated from all Swiss-Prot sequences shows that InterProScan 6 reproduces InterProScan 5 results with near-identical precision and sensitivity across all InterPro member databases. InterProScan 6 therefore enables more efficient, flexible, and reproducible genome-scale protein function annotation.

**Availability and implementation:**

InterProScan 6 is distributed under the Apache 2.0 license. Its documentation is hosted on ReadTheDocs (https://interproscan6.readthedocs.io/) and its source code is available on GitHub (https://github.com/ebi-pf-team/interproscan6).

## 1 Introduction

The rate of sequencing novel biological constructs far exceeds our capacity for accurate laboratory-based function annotation. Consequently, the function of most protein sequences is poorly defined, and computationally assigning function to protein sequences is a fundamental task in bioinformatics. InterProScan (Jones *et al*. 2014) is a tool designed to scan protein sequences against a diverse array of predictive models, ranging from hidden Markov models (HMMs) to BLAST-based searches, with the aim to detect domains, motifs, and other sequence features. Following the initial search, InterProScan conducts post-processing of the raw outputs, filtering, and integrating the results with the corresponding entries in the InterPro database (Blum *et al*. 2025). This not only establishes the presence of specific sequence signatures but also enriches the functional annotations with additional data such as Gene Ontology (Gene Ontology Consortium *et al.* 2023) terms and pathway-related information. This strategy of model-driven scanning and systematic annotation has made InterProScan integral to numerous large-scale genome annotation projects and bioinformatics resources, including UniProt (UniProt Consortium 2025), Ensembl (Dyer *et al*. 2025), and MGnify Genomes (Gurbich *et al*. 2023).

InterProScan 5, the current implementation of InterProScan, represented a significant advance in the software’s evolution, providing an architecture to coordinate complex analyses across compute resources. However, several limitations became apparent as the scale and complexity of bioinformatics workflows grew. Notably, the application was tightly coupled to specific data releases, requiring users to download large bundles containing the binaries and all signature data, regardless of their specific needs. Dependencies such as Perl and Python required separate management, and integration with modern workflow engines was cumbersome. Additionally, although the match lookup service, a web interface for retrieving pre-calculated matches, was technically functional, it was not designed for use outside of InterProScan itself. Its response format, an XML document containing CSV-formatted match data, made it difficult to use programmatically and discouraged its adoption by the wider scientific community, although the service could have provided convenient access to InterPro annotations for any sequence in UniParc.

To address these challenges and by leveraging advancements in workflow management and containerisation, we have developed InterProScan 6. This new version is a complete reimplementation built using the Nextflow workflow management system (Di Tommaso *et al*. 2017). InterProScan 6 enhances scalability, flexibility, ease of use, and reproducibility, incorporating updated analysis tools, and improved data handling mechanisms.

## 2 Methods

InterProScan 6 introduces several major changes and new features compared to its predecessor.

### 2.1 Expanded system support

**Figure 1:**
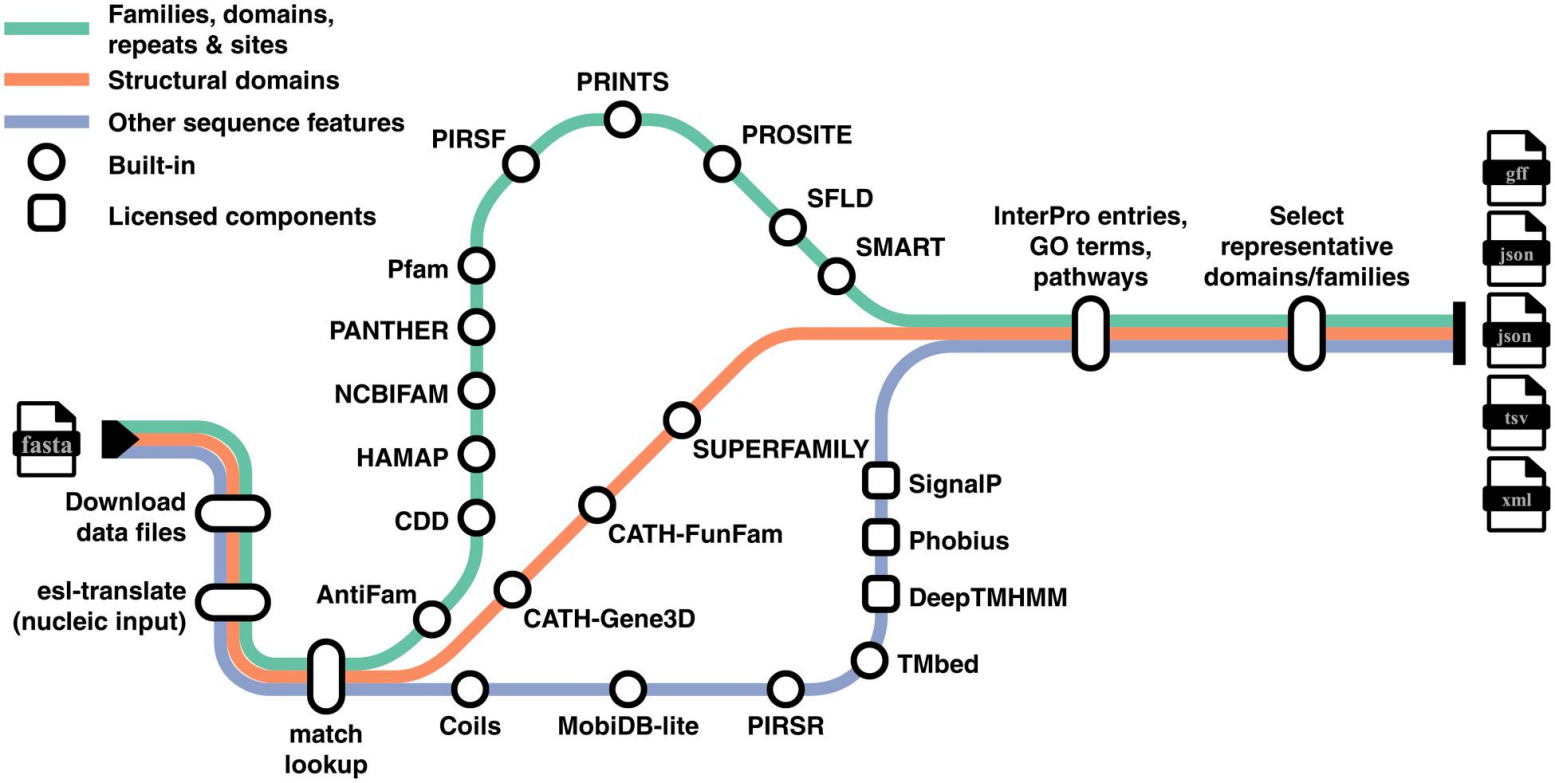
Overview of the InterProScan 6 analysis pipeline. Protein sequences are analysed through a model workflow combining member database searches, sequence feature prediction, and post-processing steps to generate integrated InterPro annotations. Built-in and licensed components are indicated. Member databases are grouped by annotation type, not by execution order. Where available, pre-computed annotations are retrieved via checksum-based lookup against the Matches API, allowing local analyses to be skipped. Final outputs are produced in standard formats, including JSON, GFF3, TSV, and XML.

The core of InterProScan 6 is its implementation as a Nextflow pipeline. Nextflow provides robust workflow execution, parallelisation, and implicit scalability across local machines and distributed compute systems such as Slurm, LSF, and AWS. Nextflow’s execution model also enhances reproducibility by managing processes and their dependencies. Unlike InterProScan 5, which is executed as a standalone application on Linux hosts with optional Docker support, InterProScan 6 is implemented as a native workflow and supports Windows, Linux, MacOS and the Slurm cluster scheduler, as well as Docker, Singularity and Apptainer out of the box. Users can also extend compatibility to any system or container runtime supported by Nextflow by creating custom execution profiles. InterProScan 6 can also be deployed without a standardized or shared compute environment. In such cases, users can run the workflow on their own workstation or virtual machine with Nextflow and a supported container runtime, while managing storage and compute resources locally. The overall structure of the InterProScan 6 workflow is shown in Fig. 1.

### 2.2 Improved scalability

The new architecture also addresses scalability requirements for analysing millions of sequences. SMART (Letunic *et al*. 2021) profile HMMs were originally created using HMMER2 and, unlike models from other InterPro member databases, have not been updated to HMMER3. Consequently, InterProScan 5 relied on HMMER2 for SMART searches. HMMER3 provides substantially improved performance over HMMER2 whilst maintaining high sensitivity (Eddy 2011). Accordingly, InterProScan 6 adopts a two-stage search strategy for SMART analyses in which protein sequences are first screened using HMMER3-converted models to identify candidate hits, after which HMMER2 searches are restricted to the corresponding SMART models. This approach reduces processing time whilst preserving the SMART matches historically reported by InterProScan 5. Additionally, pre-screening for potential CATH-FunFam matches using CATH-Gene3D has been reconfigured in InterProScan 6, such that enabling CATH-Gene3D and CATH-FunFam searches no longer requires running the CATH-Gene3D search twice, thus reducing computational time and resource requirements.

### 2.3 Updated analysis tools

InterProScan 6 discontinues support for TMHMM (Krogh *et al*. 2001) and SignalP 4.1 (Petersen *et al*. 2011), which relied on older statistical models that are no longer maintained and show reduced performance compared to current deep-learning approaches. InterProScan 6 adds optional support for DeepTMHMM (Hallgren *et al.* 2022) and SignalP 6 (Teufel *et al*. 2022) for transmembrane-topology prediction and signal-peptide detection when installed separately as their code and models cannot be redistributed. Differences in predicted transmembrane regions or signal peptides arise solely from the use of updated prediction models and not from changes in downstream integration.

TMbed (Bernhofer and Rost 2022) is introduced as the fully integrated, default method for identifying transmembrane regions and signal peptides, and its licensing terms allow distribution with InterProScan.

Deep-learning-based tools run on CPU by default, but can make effective use of GPUs to reduce runtime. InterProScan 6 provides a built-in command-line option (−use-gpu) to enable GPU-accelerated execution for these tools. This is supported on HPC systems such as Slurm or LSF, as well as on AWS Batch and Google Batch, provided that the underlying queues or compute environments are configured to include GPU-capable instances.

All member database analyses (e.g. Pfam, CDD, PANTHER) continue to use their standard search engines (e.g. HMMER, BLAST), and PHOBIUS (Käll *et al*. 2004) continues to be supported, providing combined prediction of signal peptides and transmembrane topology.

To maintain backward compatibility, InterProScan 6 produces JSON output that closely follows the structure and content of the InterProScan 5 JSON format, with only minor differences that may require limited adjustments in downstream parsers.

### 2.4 Decoupled application and data management

A significant architectural change is the decoupling of the InterProScan application code from the member database signature files. In InterProScan 5, each release distributed the application together with a muti-gigabyte data bundle of required data files, meaning every version included its own full copy of the data. By separating the pipeline code from the underlying signature data InterProScan 6 allows multiple versions of InterPro to coexist within a shared, centralised repository of data files, thus avoiding redundant storage of unchanged data. Users can specify the desired InterPro data version at runtime via the –*interpro <version>* argument, and the pipeline automatically loads the appropriate metadata and selects the correct member database files. This approach brings clear advantages in shared environments such as HPC systems, where a central deployment serves many users and can save substantial disk space. It also simplifies reproducibility and maintenance: different InterPro versions can be used side-by-side without duplicating datasets, enabling exact re-execution of historical analyses by pinning both the pipeline version and the InterPro data release, a requirement for long-running genome projects.

### 2.5 On-demand data download

To streamline setup and reduce storage requirements, InterProScan 6 introduces automatic on-demand data retrieval. InterProScan 5 required users to pre-download a complete multi-gigabyte data bundle, however, InterProScan 6 automatically detects and downloads any missing signature data files needed for a given analysis. When users specify the desired applications (via –*applications <list>*) and InterPro version (*–interpro <version>*), the pipeline downloads only the necessary data before execution. Downloaded files are stored for future use so each file is retrieved only once. This significantly lowers the entry barrier for new users and enables more efficient, targeted deployments, particularly for those running only a subset of analyses.

### 2.6 Containerisation support

InterProScan 6 is designed with containerisation in mind. Whilst InterProScan 5 required host-level installation of dependencies, including Python and Perl, InterProScan 6 provides configuration profiles for Docker, Singularity, and Apptainer. This allows each step of the pipeline to run within a container that encapsulates all required binaries and software dependencies. Users need only install Nextflow and a compatible container engine, greatly simplifying setup and ensuring a consistent execution environment across different systems.

### 2.7 Revamped matches API

InterProScan 5 uses a match lookup web service to retrieve pre-calculated results for known sequences identified by checksum, avoiding redundant computation. However, this service had limitations: multiple endpoints with unclear distinctions, an undocumented XML response format containing CSV strings, and missing InterPro entry mappings.

With InterProScan 6, we introduce the new Matches API (www.ebi.ac.uk/interpro/matches/api/). This service offers a single, streamlined RESTful endpoint with Cross-Origin Resource Sharing (CORS) support and allows querying up to 100 sequence checksums per request. This per-request limit was chosen to ensure rapid response times, and in practice, even for large proteomes, sequential batching of 100 checksums adds negligible overhead relative to local analysis time. This API returns pre-computed InterPro annotations for UniParc sequences, allowing users to bypass local computation when results are already available. InterProScan 6 integrates this service directly into the execution workflow, resolving known sequences via checksum lookup prior to launching local analyses. For InterPro release 108.0 (January 2026), the Matches API exposes 12.9 billion annotations across more than one billion UniParc sequences, providing extensive coverage of publicly available protein sequences.

Users requiring larger requests or local control can deploy the Matches API server and data on their own infrastructure and configure InterProScan to query that instance. The JSON response format produced by the Matches API is identical to the JSON output generated by InterProScan 6 itself. This enables lightweight annotation workflows and facilitates reuse of InterPro annotations in external pipelines, web services, or large-scale comparative analyses without local installation of InterProScan.

## 3 Results

### 3.1 Runtime performances

We evaluated the performance of InterProScan 6 against InterProScan 5 using nine reference proteomes from UniProt representing widely model organisms spanning bacteria, fungi, plants and animals (Table 1). Across all datasets, InterProScan 6 achieved substantial reductions in wall-clock runtime compared to InterProScan 5. The largest absolute improvements were observed for complex eukaryotic proteomes such as *Homo sapiens* and *Mus musculus*, where runtimes were reduced by approximately 40% relative to InterProScan 5.

**Table 1:** Runtime comparison of InterProScan 5 (IPS 5) and InterProScan 6 (IPS 6) across nine reference proteomes.

Organism UniProt Proteome ID	Protein sequences	IPS 5	IPS 6	IPS 6 + match lookup
***A. thaliana*** **UP000006548**	27 448	9 h 29 min	6 h 10 min	0 h 05 min
***C. elegans*** **UP000001940**	19 824	5 h 30 min	3 h 49 min	0 h 06 min
***D. melanogaster*** **UP000000803**	13 822	5 h 59 min	4 h 14 min	0 h 06 min
***E. coli*** **UP000000625**	4402	1 h 29 min	0 h 39 min	0 h 02 min
***H. sapiens*** **UP000005640**	20 659	12 h 13 min	6 h 42 min	0 h 14 min
***M. musculus*** **UP000000589**	21 803	11 h 33 min	6 h 41 min	0 h 13 min
***M. tuberculosis*** **UP000001584**	3996	1 h 39 min	0 h 44 min	0 h 02 min
***S. cerevisiae*** **UP000002311**	6065	2h 54 min	1 h 22 min	0 h 03 min
***Z. mays*** **UP000007305**	39 209	10 h 51 min	7 h 28 min	0 h 05 min

Wall-clock runtimes are shown for InterProScan 5, InterProScan 6, and InterProScan 6 with checksum-based match lookup enabled. Reported runtimes correspond to the median of five independent runs for each proteome. InterProScan 5 was executed using the standalone distribution (version 5.77–108.0). InterProScan 6 (version 6.0.0) was run via Nextflow with Singularity containerised execution. All runs used InterPro release 108.0. Reference proteome sequences were obtained from UniProt (release 2025_04), using the “one protein sequence per gene” FASTA files. Benchmarks were performed on the EMBL-EBI Slurm-based HPC system using eight CPU cores per task (Intel Xeon Gold 6252), with 90 GB of memory requested per job. Runtimes reflect full pipeline execution but exclude one-time data downloads. Across all proteome and condition combinations, the relative median absolute deviation (MAD/median) across five runs was below 6%, indicating limited run-to-run variability. Individual runtimes for all benchmark executions are provided in Supplementary Table 1, available as supplementary material at *Bioinformatics Advances* online.

When the Matches API was enabled, sequences were resolved via checksum-based retrieval of pre-computed InterPro annotations for UniParc sequences, reducing end-to-end runtime to less than 15 minutes even for large proteomes. This demonstrates that annotation reuse can reduce most computational costs for characterised proteomes, whilst producing output that remains compatible with standard InterProScan formats.

### 3.2 Cloud deployment baseline

To complement the Slurm benchmarks, we evaluated InterProScan 6 on the human proteome in two cloud deployment modes on AWS and Google Cloud. In the first, the workflow was executed on a single dedicated instance with local storage; in the second, a hybrid configuration was used in which input and output files remained local while compute-intensive tasks were dispatched through AWS Batch or Google Batch. Single-instance execution cost $10.14 on AWS and $8.13 on Google Cloud for the full human proteome.

Hybrid execution increased parallelism by distributing tasks across multiple instances and reduced wall-clock time to less than two hours, while keeping total cost reasonably close to the single-instance runs at $12.43 on AWS and $11.53 on Google Cloud. For hybrid runs, reference data required by the workflow (e.g. HMM profiles) were pre-staged in cloud object storage, so these measurements exclude initial data download and staging. Total cost does not correspond directly to wall-clock time multiplied by the quoted hourly rate, because charges accumulate across multiple concurrently running instances, with additional overhead from transient file transfers.

These hybrid runs used spot instances, which reduce compute cost relative to on-demand instances but may be terminated by the cloud provider. In such cases, InterproScan 6 resubmits the affected tasks and because input FASTA files are split into smaller chunks, most tasks remain short-lived, limiting the impact of preemption; spot-instance termination is mainly disadvantageous for tasks that run for several hours. Spot instances are not enabled by default: users should refer to the Nextflow documentation for cloud-specific configuration, noting that this is a one-line setting for Google Batch, while AWS Batch requires a compute environment configured to use Spot capacity.

### 3.3 Annotation concordance between InterProScan 5 and InterProScan 6

To assess whether the reimplementation affected annotation results, we compared annotations produced by InterProScan 5 and InterProScan 6 across the entire Swiss-Prot dataset (release 2025_04; 573,661 protein sequences). InterProScan 5 annotations were treated as the reference set, and agreement was evaluated independently for each InterPro member database at the match level using standard definitions of precision (TP/(TP+FP)), sensitivity (TP/(TP+FN)), and F1 scores as the harmonic mean of precision and sensitivity, where true positives correspond to matches reported by both versions under the overlap criterion (Table 2). Two annotations were considered equivalent when their match boundaries overlapped reciprocally by at least 80%.

**Table 2:** Concordance of annotations produced by InterProScan 5.77–108.0 and InterProScan 6.0.0 on Swiss-Prot sequences.

Member database	Precision	Sensitivity	F1 score
**CDD**	1.00	1.00	1.00
**CATH-Gene3D**	0.998	0.999	0.998
**HAMAP**	1.00	1.00	1.00
**NCBIFAM**	1.00	1.00	1.00
**PANTHER**	0.985	0.999	0.992
**Pfam**	1.00	1.00	1.00
**PIRSF**	0.999	0.999	0.999
**PRINTS**	1.00	1.00	1.00
**PROSITE Patterns**	0.999	0.999	0.999
**PROSITE Profiles**	1.00	1.00	1.00
**SFLD**	1.00	1.00	1.00
**SMART**	0.999	0.993	0.996
**SUPERFAMILY**	1.00	1.00	1.00

Precision, sensitivity, and F1 scores are reported for each InterPro member database based on scans of the full Swiss-Prot database (release 2025_04). InterProScan 5 annotations were treated as the reference set, and agreement was evaluated at the match level. Two annotations were considered equivalent when their match boundaries overlap reciprocally by at least 80%.

Across all member databases, InterProScan 6 showed near-perfect agreement with InterProScan 5. For most databases, precision and recall were equal to 1. Minor deviations were observed for a small number of databases, but F1 scores remained above 0.99. These differences are attributable to changes in execution strategy and updated tool implementation rather than changes to search parameters.

## 4 Conclusion

InterProScan 6 represents a complete modernisation of the InterProScan framework, offering a scalable, workflow-native implementation. By combining a Nextflow-based architecture, container-first execution, decoupled data management, and seamless reuse of pre-computed annotations by the Matches API, InterProScan 6 substantially improves performance, reproducibility, and ease of deployment. Benchmarking demonstrates that these architecture changes achieve significant runtime reductions while preserving annotation consistency with InterProScan 5. InterProScan 6 provides a sustainable foundation for large-scale protein function annotation in contemporary computation environments.

## Supplementary Material

vbag141_Supplementary_Data

## Data Availability

InterProScan 6 is distributed under the Apache 2.0 license. Its documentation is hosted on ReadTheDocs (https://interproscan6.readthedocs.io/) and its source code is available on GitHub (https://github.com/ebi-pf-team/interproscan6).
